# An interview with **Carlos Alberto Estevanell Tavares**

**DOI:** 10.1590/2177-6709.20.5.018-027.int

**Published:** 2015

**Figure:**
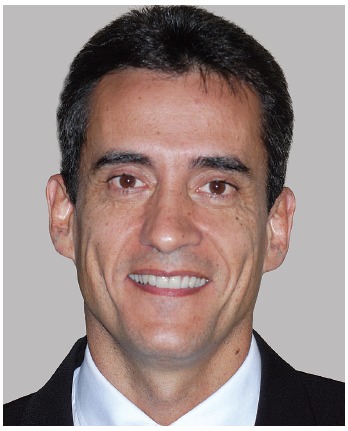

Dr. Carlos Alberto Estevanell Tavares was born in Porto Alegre in 1960. His father, Armando Petersen Tavares, was a pioneer of Brazilian Orthodontics and obtained his degree from Columbia University in the city of New York where he met his wife Luisa Estevanell from Cuba. Carlos Alberto attended the traditional Colégio Farroupilha, a high school in the city of Porto Alegre. He graduated with a degree in Dentistry from Universidade Federal do Rio Grande do Sul (1983) and followed his father's steps by obtaining postgraduate, Master's and PhD degrees in Orthodontics from Universidade Federal do Rio de Janeiro. He is married to Beatriz, a cheerful and high-spirited person, with whom he has two children: Bárbara and Bruno. In 2004, he took his first Brazilian Board of Orthodontics and Facial Orthopedics (BBO) exam and received a certificate of excellence in Orthodontics and Facial Orthopedics. Presently, he is BBO president-elect and professor of the postgraduate program in Orthodontics at ABO/RS. He has several articles published in national and international scientific journals. Carlos Alberto is highly respected and admired by his colleagues for his probity of character, earnest and ability in conducting orthodontic treatment. For this reason he is invited to give courses and lectures all over Brazil. 

O Dr. Carlos Alberto Estevanell Tavares nasceu em Porto Alegre, em 1960. Seu pai, Armando Petersen Tavares, foi um dos pioneiros da Ortodontia no Brasil, tendo feito sua formação na Universidade de Columbia, em Nova York, onde conheceu sua futura esposa, Luisa Estevanell, natural de Cuba. Carlos Alberto estudou no tradicional Colégio Farroupilha de Porto Alegre. Formou-se em Odontologia na Universidade Federal do Rio Grande do Sul (1983) e, posteriormente, seguiu os passos do pai, completando sua formação na especialidade de Ortodontia, com mestrado e doutorado na Universidade Federal do Rio de Janeiro. É casado com Beatriz, pessoa alegre e vibrante, com quem tem dois filhos: Bárbara e Bruno. Em 2004 submeteu-se ao primeiro exame do Board Brasileiro de Ortodontia e Ortopedia Facial (BBO), recebendo a certificação de Excelência na prática da Ortodontia e Ortopedia Facial. Atualmente, é presidente eleito do BBO e professor do Curso de Especialização em Ortodontia da ABO/RS. Possui inúmeros artigos publicados em revistas científicas nacionais e internacionais. É respeitado e admirado por seus colegas pela retidão de conduta, seriedade e competência na condução de seus tratamentos. Por conta disso, é convidado a ministrar cursos e palestras em todas as regiões do Brasil. 

Luciane Macedo de Menezes

**Undoubtedly, you are a highly respected and admired professional. If you look back, what are the factors you consider to be key to build such a professional profile?**(Cátia Quintão)

I appreciate your kind words. I have always tried to follow the lessons and especially the professional principles given by the professors of the postgraduate program I attended at Universidade Federal do Rio de Janeiro: the ongoing quest for excellence in orthodontic practice, which includes personal and ethical patient care, in addition to the ongoing up-todate specialized education achieved not only by attending congresses in Brazil as well as overseas, but also by having continuous access to high-quality specialized literature. To my view, undergoing the standard Edgewise technique training is also import to the orthodontist, since those who master it are more likely to be able to make archwire bends, necessary and unavoidable at treatment finishing, and, as a result, achieve high-quality final outcomes. I also made progress as an orthodontist from the time I sat for the BBO exam. The Brazilian Board of Orthodontics and Dentofacial Orthopedics has been key to defending high-quality ethical Orthodontics. In my opinion, specialized education is an ongoing process that must be followed throughout one's career, regardless of the field of work. Presently, new technology has rapidly arisen, and those who do not adopt such innovations run the risk of becoming obsolete.

**You were one of the first to use skeletal anchorage in Brazil. Your extensive experience in using anchorage devices might be the basis for recent graduates at the beginning of their career in Orthodontics. What is your recommendation for using mini-implants as well as mini plates? Are these devices recommended in different situations?** (Daltro Ritter and Lincoln Nojima)

As soon as mini-implants and skeletal anchorage were developed,^1^^,^ 2 it was acknowledged that an exceptional resource was being presented to orthodontists. Complex movements, such as intrusion of posterior teeth, became extremely simple with such resources. As a consequence, intrusions rendered anterior open bite treatment easier, even though anterior open bite treatment stability remain a challenge to clinicians.^3^^,^ 4 I encourage recent graduates to deepen their knowledge on the use of temporary skeletal anchorage devices. At present, they play a major role in orthodontic treatment, from more complex cases to those involving patient's lack of compliance and the use of removable appliances or intermaxillary elastics.

**Figure 1:**
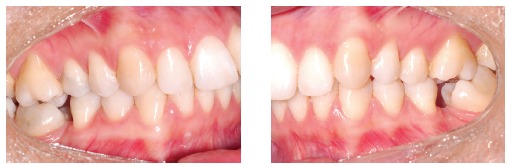
- A typical case of mini-implants use: mesialization of mandibular second and third molars performed to close spaces resulting from early first molars loss.

**Figure 2:**
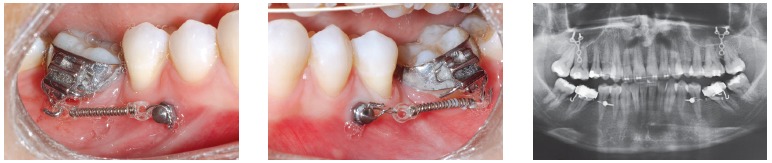
- Mini-implants placed between premolars with nickel-titanium springs and long molar hooks, so as to allow the line of mesialization force to go as near as possible the center of resistance of molars, thereby preventing further proclination.

**Figure 3:**
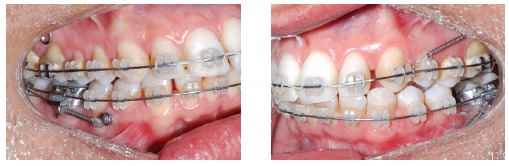
- Fixed appliance is required to control the tendency towards molar rotation.

**Figure 4:**
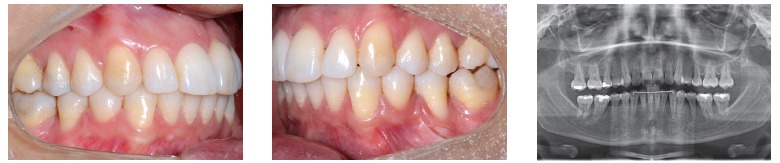
- Uprighted molars and complete space closure.

**Figure 5:**
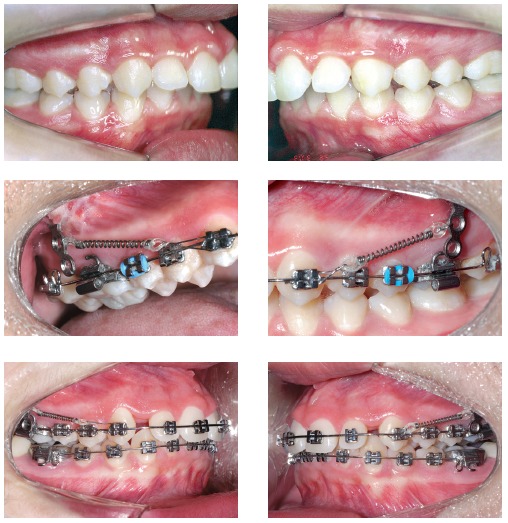
- Bilateral Class II malocclusion patient using mini plates with force vectors in horizontal or vertical direction. Within a few months (4-5), a Class I relationship is achieved.

Mini-implants offer countless possibilities of use, among which intrusion of posterior teeth and mesialization or distalization of one or more teeth in a given quadrant are highlighted. The advantages provided by mini-implants are, as follows: non invasive placement and low cost. The disadvantages, on the other hand, are: mini-implants have relatively high loss rates, do not hold too much load and are normally placed near tooth roots, which might hinder tooth movement (Figs 1 to 4).^5^ Mini plates are extremely versatile and are my favorite for large-scale movement of quadrants as a whole or when correcting anteroposterior discrepancy of great magnitude. The advantages provided by mini plates are, as follows: they are placed far from tooth roots and into basal bone and, therefore, present with loss rates near zero. Additionally, they are able to hold heavy loads and can be planned so as to be placed practically at any site in the mouth. The disadvantages, however, are: they require invasive surgical procedures and have higher costs (Fig 5).^6^

**Conventional surgical procedures of impaction of the maxilla and mandibular advancement for Class II patients with vertical excess have been discussed and associated with different approaches for impaction of the maxilla aimed at reducing the occlusal plane and decreasing the amount of mandibular advancement. What is your experience with this approach?** (Lincoln Nojima)

**Figure 6:**
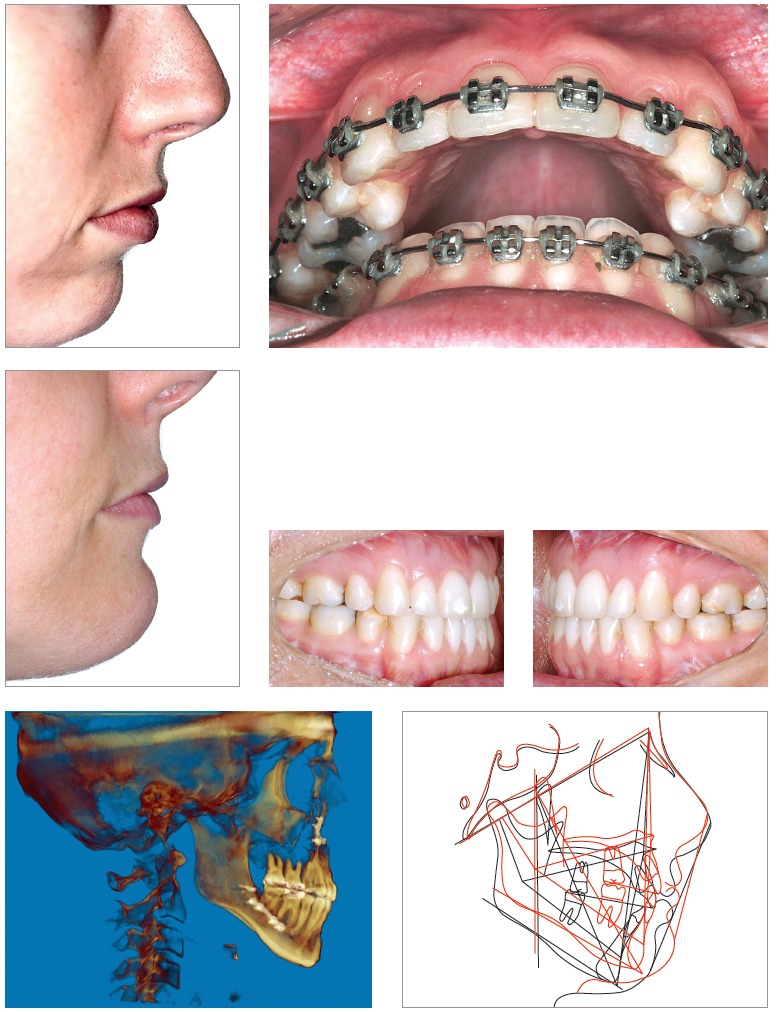
- Class II patient with vertical excess subject to impaction of the maxilla aimed at reducing the dimensions of the occlusal plane and decreasing the amount of mandibular advancement.

Facial aesthetics standards have made some progress, and slightly longer faces, showing little gingiva at smiling, are now acceptable. Thus, many cases have been currently treated with less impaction of the maxilla or clockwise rotation of the occlusal plane (contrary to counterclockwise rotation sometimes recommended to project the mandible) associated with advancement genioplasty, so as to counterbalance lack of or little mandibular advancement. Nevertheless, this approach should not be the general rule, but an option individually applied to patients under favorable conditions.^7^ Many cases also present with maxillary transverse deficiency which should be diagnosed and properly treated without further compensations. With tridimensional evaluation of the facial skeleton and joints, and thorough clinical examination, orthodontists and surgeons are able to custom treatment planning according to each patient's needs while seeking stable functional outcomes without causing any damages or overload to the joints and achieving satisfactory esthetics (Fig 6).

**With the use of new imaging diagnostic tools, such as cone-beam computed tomography associated with digital models, virtual orthognathic surgery has been increasingly performed. What is your opinion about this new surgical approach?**(Lincoln Nojima) 

**Has it changed your way of planning orthosurgical cases? Do you have a preference for any particular software? Why? **(Daltro Ritter)

All diagnostic and planning tools are welcomed. To my view, clinical examination and facial aesthetics evaluation carried out by orthodontists and oral and maxillofacial surgeons, as well as by the patient, are paramount to decision making. Patients who are in need of orthosurgical treatment are provided with a questionnaire with objective questions and answers on dental, facial and functional aspects, so that they are able to provide us with a self-analysis about their own face and teeth. With these data in hand, we can fully understand patient's expectations and, therefore, present potential solutions. Digital diagnosis has advantages, such as reduced planning time and easy virtual communication. Additionally, it might be used for surgical guide manufacture, which eliminates the need for a series of steps likely to lead to manufacturing errors. I also consider these advantages to be even clearer when treatment planning includes previous orthognathic surgery, anticipated surgery or anticipated benefit. Dolphin is the system I am most familiarized with, as it is used by the oral and maxillofacial surgeons I work with. Due to providing clear digital images, treatment issues are easily evinced. Presently, I believe the ideal is that professors, with good clinical experience and technical knowledge, use digital tools in their practice as well as to teach residents and postgraduates. Digital tools do not replace one's knowledge and experience, but are key to supplement treatment planning, communication and long-term follow-ups of finished cases.

**You have extensive experience in treating adult patients. Do you believe they have been greatly concerned about the esthetic features of appliances? Which systems do you use for esthetic appliances? And which one provides adult patients with the best cost-benefit relationship? **(Cátia Quintão) 

**Do you believe adult patients' requirements for less extraction and more esthetic material have influenced the orthodontic practice? **(Daltro Ritter)

Undoubtedly, the demand for orthodontic treatment by adult patients has increased. However, orthodontic treatment is associated with the misconception that it is only aimed at children and adolescents; for this reason, further alternatives to replace conventional metal appliances have been sought. In this context, the three most discreet alternatives to metal appliances are: ceramic brackets, lingual brackets and plastic aligners. As regards ceramic brackets, I believe they are the only ones yielding satisfactory outcomes, in comparison to plastic, glass fiber and polycarbonate ones which undergo changes in color as well as shape, in addition to having high friction rates.^8^^,^ 9 Lingual brackets are superior in terms of esthetics, but provide a number of complications, namely: difficulty being viewed by the orthodontist, patients greater discomfort, and hindered hygiene and speech.^10^^,^ 11 ^,^ 12 Plastic aligners are extremely limited, especially in terms of quality of treatment completion.^13^ Additionally, their use is limited to less complex cases. As for tooth extraction for orthodontic purposes, in the last few years, there has been indeed a tendency towards non extraction treatment, particularly due to pressure exerted by dentists referring patients to treatment, pediatric dentists and adult patients who feel uncomfortable with the idea of extracting any teeth and, therefore, require other alternatives.^14^ Interproximal enamel wear under high speed (stripping) has been one of the options to reduce the number of tooth extractions.^15^^,^ 16 With the advent of skeletal anchorage temporary devices, another feasible alternative to tooth extraction, particularly of mandibular premolars and incisors, emerged. In spite of that, third molars often end up being referred for extraction, an alternative often preferred by patients for esthetic reasons, especially because third molar extraction is not visible.

**Orthodontic treatment planning of adult patients often requires a multidisciplinary approach. How do you plan such cases?** (Luciane Menezes)

**Figure:**
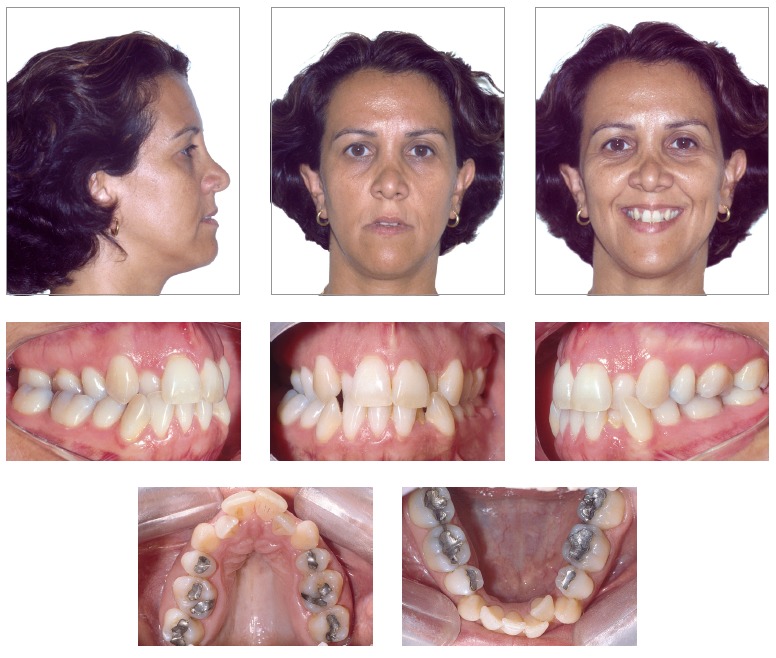

I usually highlight to my students that orthodontic treatment of adult patients, even those with health dentition, requires at least two specialties: Orthodontics and Periodontology. Periodontal disease in young patients is limited to inflammation of the protection periodontium, whereas adult patients have more significant bone loss. Orthodontic treatment does not lead to bone loss; however, whenever it is associated with periodontal disease, it speeds loss up.^17^^,^ 18 Most cases involving adult patients include a number of specialists: implant dentists, endodontists, periodontists and prosthetists. In these cases, it is paramount that all professionals get together with the patient and require all the necessary records, so as to establish a complete treatment planning that includes the sequence in which procedures will be carried out, for instance: 1^st)^ periodontal health recovery; 2^nd)^ endodontic treatment; 3^rd)^ caries and impaired restoration removal; 4^th)^ implant placement with provisional prostheses for subsequent anchorage; 5^th)^orthodontic treatment; 6^th)^ esthetic procedures; and 7^th)^permanent prostheses placement.^19^

Cases of orthognathic surgery require meetings with the oral and maxillofacial surgeon to be scheduled more frequently; before orthodontic treatment onset, before surgery, during surgery (I am usually present at the time of surgery) and before postsurgical orthodontic treatment onset. It is extremely important that the orthodontist and the surgeon try to prevent disagreement on their opinions, since it might cause the patient to feel very insecure. 

**Based on your experience, what are the major difficulties orthodontists face in orthosurgical treatment?** (Luciane Menezes)

Orthognathic surgery has been used for many years as an effective means to correct complex dental facial deformities; however, knowing about specific orthodontic biomechanics is paramount to achieve the desired orthosurgical result. Treatment combining Orthodontics and orthognathic surgery has a number of specific characteristics that range from facial analysis to orthodontic mechanics which oftentimes differs considerably from conventional mechanics applied to non surgical cases. Orthodontists willing to treat patients requiring orthognathic surgery as an important part of treatment should be aware of up-to-date surgical techniques and their effects produced on patient's face. Such knowledge is essential when proposing a treatment planning that meets patient's expectations. 

**Figure 7:**
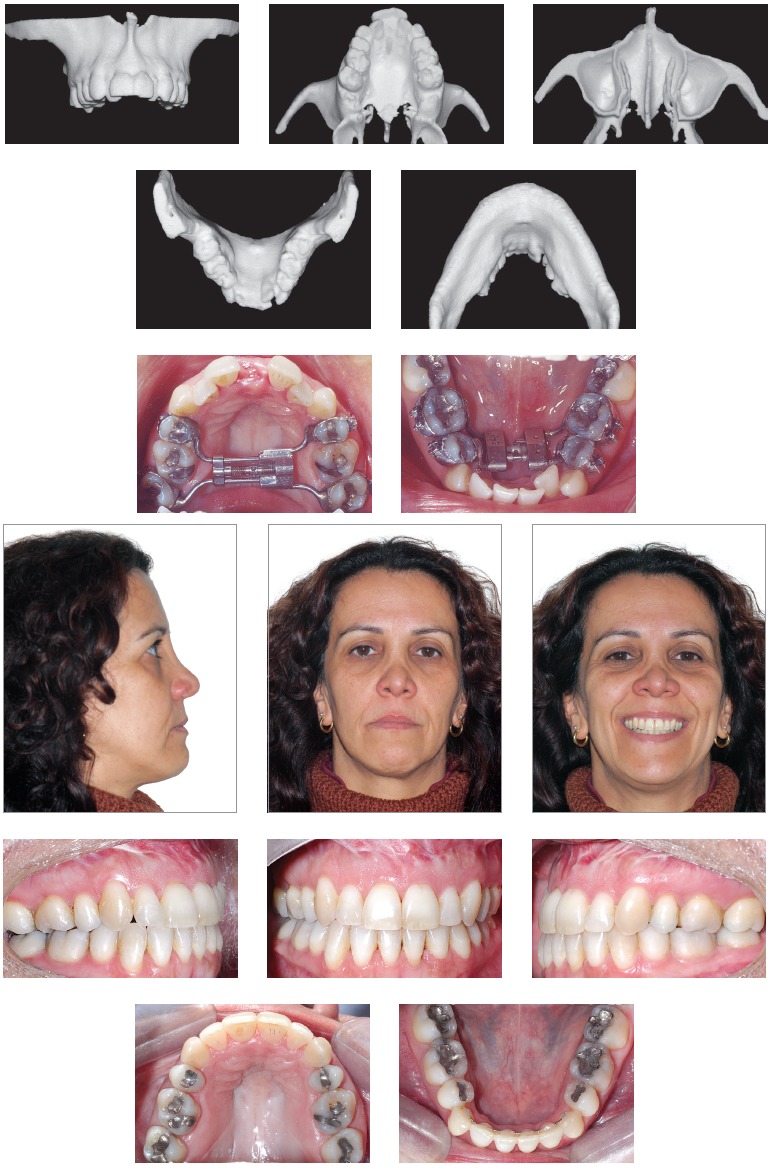
- Patient subject to orthodontic treatment with the aid of tooth-borne distractors in the maxilla and the mandible. Significant improvement in the buccal corridor is evinced.

New orthognathic surgery procedures have been recently developed, including distraction osteogenesis and anticipated surgery as well as anticipated benefit. Distraction osteogenesis is a biological process used to disconnect and handle two bone surfaces with a view to widening or expanding the bones of the arches. It has been used for both the maxilla and the mandible. Placing the appliance while determining the center of resistance of osteotomy segments and the application of forces is paramount to minimize potential complications and improve treatment outcomes (Fig 7).^20^

At present, orthodontists have many treatment options that aid them to achieve their objectives. In general, all options aim at achieving the same orthodontic outcomes: Class I occlusion. Nevertheless, each treatment option somehow affects facial aesthetics, which is more significant in cases combining orthodontic treatment and orthognathic surgery.^21^ Deep knowledge of the effects produced by surgical procedures not only on the face, but also on the teeth, is crucial for the orthodontist to prepare a predictable and reliable treatment planning.

**What are your expectations towards Brazilian Orthodontics? Do you believe it is possible to be a winner in this new scenario? **(Cátia Quintão)

I believe there will always be a place for highquality work in any field of study. The Brazilian Board of Orthodontics and Facial Orthopedics has supported this cause for over ten years, and has brought together orthodontists who also believe so. To my view, those who take orthodontic treatment quality above everything should join the BBO to fight for this cause.
